# An evidence-based approach to pericardial synovial sarcoma: a unique case report

**DOI:** 10.3332/ecancer.2025.2000

**Published:** 2025-09-25

**Authors:** Elias Zonana-Schatz, Jenniffer Ann-Swain, Jenny Naomi Shiraishi-Piña, Marcos Cherem-Kibrit, José Rodrigo Espinosa

**Affiliations:** 1 Department of Internal Medicine, Hospital de Beneficiencia Española, Mexico City 52780, Mexico; 2 Department of Cardiology, Hospital de Beneficiencia Española, Mexico City 11550, Mexico; 3 Faculty of Health Sciences, Anáhuac University, Mexico City 52786, Mexico; 4 Department of Oncology, Hospital de Beneficiencia Española, Mexico City 11520, Mexico; a https://orcid.org/0009-0000-9341-2829; b https://orcid.org/0000-0001-5348-7885

**Keywords:** pericardium, synovial sarcoma, chemotherapy, immunohistochemistry, genetic testing

## Abstract

Synovial sarcoma is a rare and aggressive mesenchymal neoplasm characterised by the presence of the SS18-SSX fusion oncogene, resulting from the chromosomal translocation t(X;18)(p11.2;q11.2). Although these tumours typically arise in the extremities, they have also been documented in atypical locations such as the pericardium, underscoring their versatile and aggressive nature. This case involves a 46-year-old male who presented with a 2-month history of neck and precordial chest pain, ultimately diagnosed with a biphasic synovial sarcoma of the pericardium. Initial imaging studies, including magnetic resonance imaging and transthoracic echocardiogram, revealed a large encapsulated intrapericardial mass with hemorrhagic and thrombotic components, severe pericardial effusion and biventricular dysfunction. Histopathological examination confirmed the diagnosis, with immunohistochemistry findings positive for CKAE1/AE3, TLE-1, EMA, BCL-2 and CD99, along with a proliferation index of 40%. The chemotherapy regimen of ifosfamide, mesna and doxorubicin proved effective for this condition, leading to a significant reduction in tumour size and metabolic activity. However, due to disease recurrence and the presence of a KDM5A-positive marker, second-line therapy with trabectedin and pazopanib became necessary.

## Introduction

Synovial sarcoma is a rare malignant mesenchymal neoplasm that typically arises in the extremities but can occasionally present in unusual locations, such as the pericardium [1]. These tumours are primarily defined by the SS18-SSX fusion oncogene, resulting from a t(X;18)(p11.2;q11.2) translocation [2, 3]. Fewer than 15 cases of pericardial synovial sarcoma have been documented in the last decade [4], highlighting its rarity and the diagnostic challenges it poses. Clinically, these tumours may present with symptoms such as chest discomfort, pericardial effusion or cardiac dysfunction and their aggressive behaviour often leads to poor outcomes.

This report highlights a biphasic pericardial synovial sarcoma in a 46-year-old man who presented with chest pain in the precordial area and was discovered to have a large mass within the pericardium.

## History of presentation

A 46-year-old male presents with a 2-month history of neck pain radiating to the back, which subsequently progressed to precordial chest pain described as oppressive, rated 4/10, without radiation. The patient sought a cardiology consultation, leading to the diagnosis of a large encapsulated intrapericardial mass with associated findings suggestive of malignancy.

An magnetic resonance imaging (MRI) revealed a large encapsulated intrapericardial mass measuring 62 mm, featuring a hemorrhagic thrombotic component, nodular pericardial thickening, severe pericardial effusion and biventricular dysfunction, all suggestive of malignancy. A transthoracic echocardiogram showed a heterogeneous intrapericardial tumour located anterior-laterally to the pulmonary artery, attached to the left ventricle, measuring 52 × 42 mm, with no pulmonary artery obstruction.

**Figure 1.**
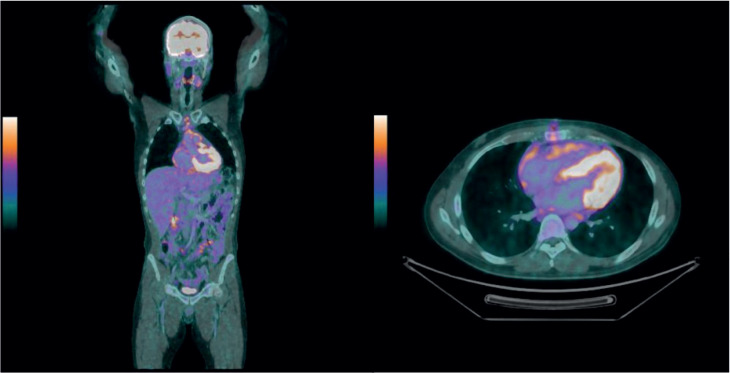
FDG PET positive for a solid lesion in the superior pericardial recess, extending to the myocardium (anterior wall of the left ventricle), as well as multiple solid nodules diffusely distributed in the pericardium and mediastinal lymph nodes with hypermetabolism.

Additional tests, such as positron emission tomography/computed tomography (PET-CT), revealed global cardiomegaly, multiple nodular areas of pericardial thickening, a solid lesion near the aortic root (62 × 27 mm) with increased metabolic activity (SUVmax 12.0) and involved lymph nodes (Figure 1).

**Figure 2.**
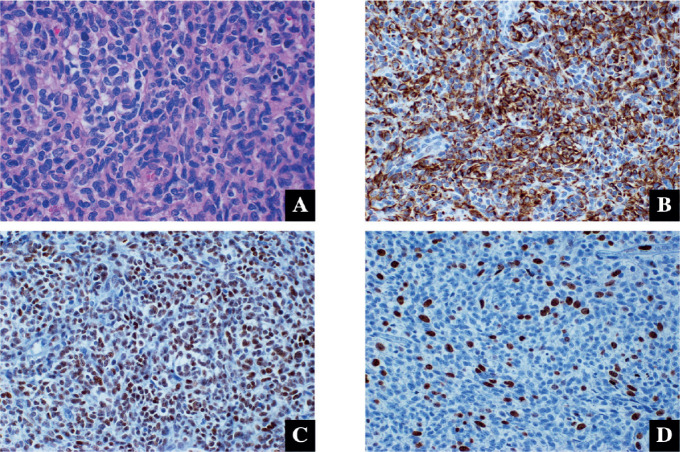
Synovial sarcoma histopathology. (a): Medium fusiform cells are observed, with oval-shaped nuclei and coarse chromatin. Hematoxylin-eosin 40×. (b–d): These cells express cytokeratins AE1/AE3 (b) and TLE-1 (c), with a proliferation index of 40% (d). 40×.

**Figure 3.**
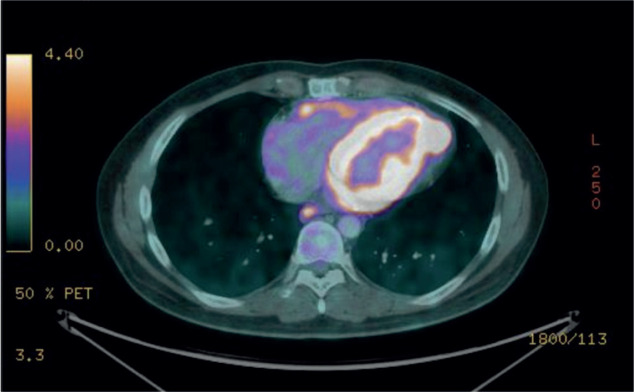
FDG PET scan with increase in both the size and metabolic tumor volume of the pericardial lesions which have heterogeneous density, predominantly hypodense, with persistent abnormal radiotracer uptake and a SULmax of 14.5 and VTM of 77.3.

The diagnosis was confirmed through histopathology studies, indicating biphasic synovial sarcoma with focal lymphovascular permeation. Immunohistochemistry results showed CKAE1/AE3+, TLE-1+, EMA+, BCL-2+ and CD99+. The proliferation index was 40% (Figure 2). Biomarker findings from next-generation sequencing revealed MicroSatellite Status-Stable (MSS) and a tumour mutational burden (TMB) of 0 muts/megabase. Additionally, genomic findings demonstrated equivocal amplifications of CCND2 and KDM5A, the CDH1 A226fs*24 mutation and the SETD2 R2109fs*38 mutation (Figure 3).

## Management

The treatment commenced with tumour resection by the cardiothoracic surgery team, resulting in a 90% reduction in tumour mass. However, due to concerns regarding cardiotoxicity, treatment was restricted to four cycles of ifosfamide, mesna and doxorubicin. This decision was further supported by echocardiographic findings, which indicated mildly reduced systolic function, with a left ventricular ejection fraction of 55% and a global longitudinal strain of −14%. Diastolic function remained normal, along with normal right heart chamber dimensions and preserved systolic function. There were no significant valvular insufficiencies, shunts, intracavitary masses or other abnormalities. Upon follow-up imaging, the patient exhibited a complete metabolic response on MRI and PET-CT.

Following the complete response, the patient was placed under surveillance. However, the patient was lost to follow-up, and after 9 months, presented with disease recurrence, evidenced by an increase in the pericardial mass size, elevated metabolic activity and worsened lymph node involvement. Second-line therapy with pazopanib combined with trabectedin was initiated, but after 4 cycles, the patient experienced local progression in the pericardium and metastasis to the brain.

### Follow up

The most recent PET-CT examination (Figure 3) showed a significant increase in both the size and metabolic tumour volume of the pericardial lesions, indicating disease progression. Given these findings and the worsening clinical course, the treatment plan included whole-brain radiotherapy and the initiation of systemic therapy with Pazopanib.

## Discussion

Synovial sarcoma is a malignant mesenchymal neoplasm characterised by aggressive growth and a significant capacity to affect a diverse array of tissues. Its defining feature is the presence of the SS18-SSX fusion oncogene, which arises from a specific chromosomal translocation (t(X;18)(p11.2;q11.2)) [2]. The SS18-SSX fusion protein functions as an aberrant transcription factor, disrupting normal gene expression and promoting tumour formation and growth [3]. The main limitation in this case is the lack of fusion analysis involving the SS18 and SSX gene family, as the receiving laboratory does not conduct testing for these genes.

While these tumours predominantly manifest in the extremities, their occurrence in alternative locations, such as the pericardium, although uncommon, has been recorded. In a series of 12,485 autopsies, an incidence rate of 0.056% for primary cardiac tumours was identified. This case underscores the versatile and aggressive nature of this particular neoplasm.

Morphologically, synovial sarcoma may present in monophasic or biphasic forms. In the monophasic type, monomorphic spindle cells dominate and it is extremely rare to observe epithelial components. In contrast, the biphasic subtype is characterised by the presence of spindle and epithelial cells in varying proportions, which may organise into glandular structures or solid nests and cords. Poorly differentiated synovial sarcomas, often defined by highly aggressive cellular proliferation with primitive features and rhabdoid morphology, present additional diagnostic challenges due to their unpredictable behaviour and worse prognosis [1].

The immunohistochemical markers and variants of synovial sarcoma are not yet fully understood. However, Bcl-2, CD99 and cytokeratin are characteristic markers commonly associated with synovial sarcomas [5].

Clinical evidence indicates that MSS and low TMB biomarkers are linked to a decreased response to anti-PD-1 immune checkpoint inhibitors, including approved therapies like nivolumab and pembrolizumab. Furthermore, no targeted therapies exist to address genomic alterations such as equivocal CCND2, the CDH1 A226fs24 mutation and the SETD2 R2109fs38 mutation [6].

The key finding in this case is the potential role of KDM5A, as cancer cells with elevated KDM5A expression are hypothesized to be more sensitive to chemotherapeutic drugs like etoposide, doxorubicin and cytarabine [7]. KDM5A induces the expression of vascular endothelial growth factor (VEGF), driving angiogenesis, oncogenic transformation and tumourigenesis. These effects can be suppressed through KDM5A knockdown, including tyrosine kinase inhibitors (TKI) that target VEGF receptors, such as Pazopanib, the only TKI approved for synovial sarcomas, exerting antitumour effects by inhibiting angiogenesis mediated by VEGF. This inhibition reduces endothelial cell proliferation by directly blocking growth-promoting receptor tyrosine kinases, such as platelet-derived growth factor receptors, fibroblast growth factor receptors and TKIs [8].

Additionally, Pazopanib can be given as monotherapy or in combination with Trabectedin, an alkylating agent that targets the DNA minor groove to initiate cytotoxic activity. Together, these therapies account for approximately 28% of second-line treatments for metastatic synovial sarcoma. A meta-analysis comparing Pazopanib and Trabectedin found that they have similar efficacy in treating metastatic synovial sarcoma, with comparable median overall survival rates but a higher overall response rate with Pazopanib [9, 10].

The recommended approach is surgery and conventional therapy with Ifosfamide, Mesna and Doxorubicin, enhanced by Trabectedin and Pazopanib, which have demonstrated favourable clinical outcomes in metastatic soft tissue sarcomas [11].

## Conclusion

This case emphasises the critical need for an accurate diagnosis, relying on advanced imaging techniques such as PET-CT and genetic markers to confirm the diagnosis and guide therapeutic decisions concerning this type of cardiac cancerous mass.

Despite an initial positive response to ifosfamide, mesna and doxorubicin, disease progression necessitated the utilisation of trabectedin in conjunction with pazopanib, thereby underscoring the significance of adaptability in treatment strategies. The suitable treatment for such cases demands a multidisciplinary approach and ongoing monitoring of the disease's dynamic nature to facilitate timely interventions aimed at enhancing patient outcomes.

## Conflicts of interest

The author(s) reported no potential conflicts of interest regarding the research, authorship and/or publication of this article.

## Funding

The author(s) did not receive any financial support for the research, authorship or publication of this article.

## Ethical approval

This study was conducted in accordance with institutional ethical standards, and written informed consent was obtained from the patient for publication of the case and accompanying images.

## Author contributions

All authors contributed to the conceptualisation, writing and critical review of this case report.

## Disclosures

The authors have reported that they have no relationships relevant to the contents of this paper to disclose.
